# Acute cholecystitis mimicking or accompanying cardiovascular disease among Japanese patients hospitalized in a Cardiology Department

**DOI:** 10.1186/s13104-015-1790-8

**Published:** 2015-12-19

**Authors:** Michishige Ozeki, Yoshihiro Takeda, Hideaki Morita, Masatoshi Miyamura, Koichi Sohmiya, Masaaki Hoshiga, Nobukazu Ishizaka

**Affiliations:** Department of Cardiology, Osaka Medical College, Takatsuki-shi Daigaku-machi 2-7, Osaka, 569-8686 Japan

**Keywords:** Cholecystitis, Acute coronary syndrome, Electrocardiographic change

## Abstract

**Background:**

Acute cholecystitis sometimes displays symptoms and electrocardiographic changes mimicking cardiovascular problems. It may also coexist with cardiovascular disorders. We analyzed the clinical characteristic of the cardiac patients who were diagnosed with acute cholecystitis during hospitalization in the cardiology department.

**Methods:**

Using the department database, we identified 16 patients who were diagnosed with acute cholecystitis during the hospitalization in the cardiology department between June 2010 and June 2014.

**Results:**

Five patients who were initially suspected to have cardiac problems (acute coronary syndrome, four patients; Adams-Stokes syndrome, one patient) owing to their symptoms were subsequently diagnosed with acute cholecystitis. Two of these patients showed electrocardiographic changes mimicking myocardial ischemia, and three tested positive for a biomarker (heart-type fatty acid binding protein) of acute myocardial injury. The 11 remaining cardiac patients were diagnosed with acute cholecystitis during their hospitalization or at the time of admission. Prolonged fasting and/or staying in an intensive care unit (ICU) may have contributed to their condition. Among these 11 patients, aortic dissection was the most prevalent underlying cardiac condition, affecting 5 patients.

**Conclusions:**

Although it is a rare condition, acute cholecystitis may coexist with or be misdiagnosed as a cardiovascular disorder. This possibility should not be overlooked in cardiac patients because a delay in treatment may result in critical complications.

## Background

Due to the similarity of symptoms, it is sometimes difficult to make a differential diagnosis between a biliary system disease, such as acute cholecystitis, and cardiovascular disorders [1–3]. Although the exact mechanisms are poorly understood, laboratory data mimicking cardiovascular disease, such as elevation of cardiac troponin [4] and changes in electrocardiogram (ECG) data suggestive of ischemic heart disease [1, 5], may also occur among patients with cholecystitis. It should also be noted that acute cholecystitis may occur among cardiac patients because congestive heart failure [6], long stays in an intensive care unit [7], and prolonged after a cardiovascular event [8] may enhance the risk of cholecystitis. We herein analyzed the clinical characteristics of patients who were diagnosed with acute cholecystitis during hospitalization in a cardiology department.

## Methods

### Study population

The study was approved by Ethics Committee at the Osaka Medical College. Between June 2010 and June 2014, 5552 patients were admitted to the cardiology department of Osaka Medical College. From the database, we identified 18 patients who were diagnosed with acute cholecystitis during their hospitalization in the cardiology department. Of these 18 patients, 2 patients did not have symptoms suggestive of de novo occurrence or worsening of cardiovascular conditions. After excluding these two patients, the remaining 16 patients were divided into two groups as follows: group 1 included 5 patients who were initially diagnosed with cardiac conditions, and in whom the signs and symptoms were actually due to cholecystitis; group 2 included 11 patients who were admitted with a cardiac condition and cholecystitis developed during their hospitalization or coexisted at the time of admission.

### Laboratory analysis

Blood samples were generally taken after an overnight fast; however, for patients admitted in an emergency, this might have not been the case. Blood data were obtained when considered to be clinically necessary, and the baseline values and the peak values during the hospitalization regarding to the hepatobiliary data (aspartate transaminase, γ-glutamyl transpeptidase, alkaline phosphatase, Total bilirubin) and markers indicating the extent of inflammation (white blood cell count and C-reactive protein), in particular, were demonstrated in the table. Positivity for heart-type fatty acid-binding protein (H-FABP) was examined by point-of-care-testing for H-FABP using Rapicheck H-FABP (Wakunaga Pharmaceutical, Osaka, Japan).

### Diagnosis of acute cholecystitis

Acute cholecystitis was diagnosed by imaging modalities that were performed owing to suspected accompanying clinical symptoms such as fever and abdominal pain, and abnormalities in hepatobiliary and/or inflammatory laboratory data. All patients underwent computed tomography (CT) and/or ultrasonography (US) that showed typical findings indicating this diagnosis, such as gallbladder distension, gallbladder wall thickening and a trilaminar appearance or biliary calculus [9]. The diagnosis of cholecystitis was made or confirmed by gastroenterology physicians.

## Results

### Symptoms and initial diagnosis in group 1

The symptoms and past cardiovascular histories of the patients who were initially diagnosed with cardiac conditions, but who actually had cholecystitis (group 1) are shown in Tables 1 and 2, and their laboratory data on admission and during hospitalization in the cardiology department are shown in Table 3.

**Table 1:** Characteristics of patients initially diagnosed with cardiac conditions but found to have acute cholecystitis (group 1)

	#01	#02	#03	#04	#05
Symptoms	Chest oppression, dyspnea	Chest oppression	Chest pain	Chest oppression	Loss of consciousness
Initial diagnosis (suspected)	ACS	ACS	ACS	ACS	Adams-Stokes attack
ECG changes on admission	Inverted or flat T in V2-6	ST depression in II, III, AVF	None (pacemaker rhythm)	None	None
Troponin T	Negative	Negative	Negative	Negative	N/A
H-FABP	Positive	Negative	Positive	Positive	N/A
Diagnosis interval, day	1	1	0	0	1
Cholelithiasis	0	1	0	1	1
Choledocholithiasis	0	0	0	1	0
Treatment	Laparoscopic cholecystectomy	Cholecystectomy	Antibiotics	Endoscopic nasobiliary drainage	Cholecystectomy

*ACS* acute coronary syndrome, *H-FABP* heart-type fatty acid-binding protein

**Table 2:** Gender, age, and body structure of study patients

Variables	Group 1, n = 5	Group 2, n = 11
Women/men	1/4	2/9
Age, years	74.4 ± 8.6	73.5 ± 8.9
Height, m	1.62 ± 0.14	1.63 ± 0.08
Weight, kg	65.4 ± 12.0	62.0 ± 11.0
Body mass index, kg/m^2^	25.0 ± 3.5	23.1 ± 3.2

P values are meant for the difference between group 1 and group 2

**Table 3:** Baseline and peak hepatobiliary data and inflammation markers during hospitalization in the cardiology department

Variables	Group 1, n = 5	Group 2, n = 11
On admission		
White blood cell count, ×10^3^/mL	9.4 (3.3–20.7)	9.3 (5.8–14.8)
Aspartate transaminase, IU/L	198 (25–293)	23 (17–81)
Alanine transaminase, IU/L	51 (24–182)	25 (14–46)
γ-Glutamyl transpeptidase, IU/L	87 (23–301)	78 (29–208)
Alkaline phosphatase, IU/L	205 (189–333)	273 (170–418)
Total bilirubin, mg/dL	0.7 (0.6–1.0)	0.8 (0.6–1.1)
C-reactive protein, mg/dL	0.10 (0.04–3.71)	3.09 (0.54–5.94)
Peak values during hospitalization in Cardiology Department		
White blood cell count, ×10^3^/mL	14.2 (3.5–23.9)	9.3 (7.3–15.5)
Aspartate transaminase, IU/L	283 (34–314)	150 (34–300)
Alanine transaminase, IU/L	54 (38–182)	67 (38–263)
γ-Glutamyl transpeptidase, IU/L	202 (50–592)	218 (33–461)
Alkaline phosphatase, IU/L	206 (193–302)	536 (261–1099)
Total bilirubin, mg/dL	1.3 (0.7–2.5)	1.7 (0.7–2.7)
C-reactive protein, mg/dL	8.85 (1.32–13.80)	6.94 (4.89–13.03)

Numbers of patients for whom there were not data for the following parameters. Data on admission;* r-GTP* 2 patients in group 1, 1 patient in group 2,* Al-p* 2 patients in group 1, 1 patient in group 2,* CRP* 1 patient in group 1. Peak data in the cardiology department:* r-GTP* 1 patient in group 1,* Al-p* 1 patient in group 1,* CRP* 1 patient in group 1

Of the five patients in group 1, four (80 %) were initially suspected to have acute coronary syndrome owing to a sensation of chest oppression (cases 1–4). Coronary angiography ruled out a coronary artery event in cases 1, 2, and 3, and it did not any significant coronary artery stenosis in these patients. Coronary angiography was not performed in case 4 because a non-cardiac event was suspected due to the elevation of hepatobiliary enzymes. The remaining patient (case 5), who claimed loss of consciousness, was initially suspected to have had an Adams-Stokes attack because of a 3-second pulse pause when the emergency services arrived.

**Fig. 1:**
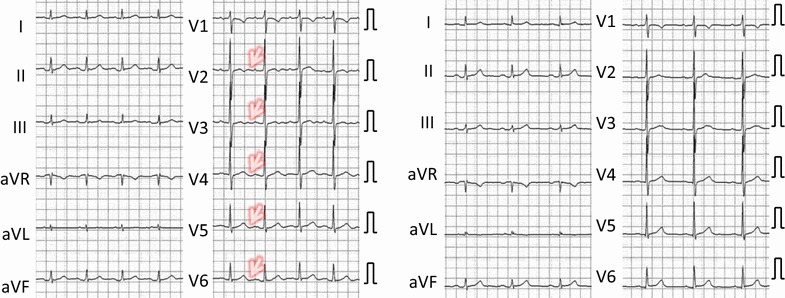
Electrocardiogram of case 1. *Left* On the admission. *Right* Five days after the admission. Changes of T wave (flat T, terminal T wave inversion) at the time of admission were not present on electrocardiogram taken after 5 days

**Fig. 2:**
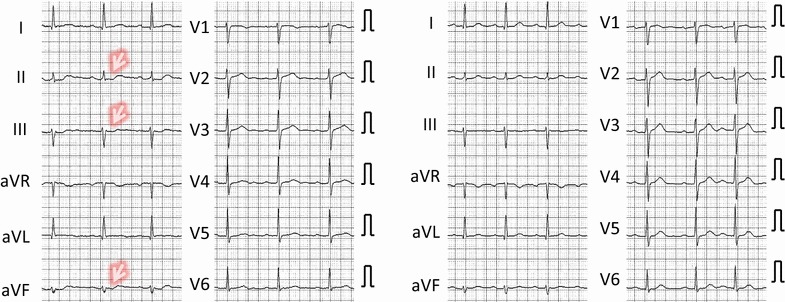
Electrocardiogram of case 2. *Left* On the admission. *Right* Ten hours after the admission. Note that ST segment depression seen in the ECG at the time of admission disappeared after 10 h

Among the four patients suspected of acute coronary syndrome, two (cases 1, 2) showed ECG changes suggestive of myocardial ischemia (Figs. 1, 2), while one did not have ECG changes (case 4). Case 3 had pacemaker rhythm; therefore, ST-T changes could not be assessed. Three of the four patients who were initially suspected to have acute coronary syndrome tested positive for H-FABP, although cardiac troponin was negative. Renal function in all patients with a positive H-FABP test was within the normal range.

Inversion of the terminal T in precordial leads and V4was observed at the time of admission in case 1 (Fig. 1, arrows). This change was not observed in ECG that was taken 5 days afterward. In case 2, ST segment depression was noted in leads II, III, and aVF (Fig. 2, arrows), but this disappeared after 10 h.

### Symptoms and initial diagnosis in group 2 and timing of the development of cholecystitis

The 11 patients in group 2 were admitted to the cardiology department with true cardiovascular conditions (Tables 2, 3, 4). The most prevalent diagnosis was aortic dissection: three patients were in the acute phase and one was in the chronic phase. The second most prevalent diagnosis was congestive heart failure.

**Table 4:** Characteristics of patients admitted with a cardiac condition who had coexisting cholecystitis or developed it during hospitalization (group 2)

Case no.	#06	#07	#08	#09	#10	#11	#12	#13	#14	#15	#16
Symptoms	Dyspnea on effort	Chest/back pain	Chest pain, fever	Abdominal pain	Chest/back pain	Clinically silent	Dyspnea on effort	Palpitation	(Post EVAR)	Dyspnea on effort	Dyspnea, fever
Initial diagnosis	Acute myocardial infarction	Acute aortic dissection (type B)	Chronic aortic dissection (type B)	Acute aortic dissection (type B)	Acute aortic dissection (type A)	Complete AV block	Congestive heart failure	Paroxysmal Af for ablation	Coronary artery disease	Congestive heart failure	Congestive heart failure, Af
Cardiovascular past histories	Post CABG	Paroxysmal Af	None	None	None	None	Atrial flutter	Myocardial infarction	Ileac artery aneurysm	Myocardial infarction	Sick sinus syndrome
Diagnosis interval, day	13	9	5	8	20	21	0	4	0	24	3
Cholelithiasis	Yes	No	No	Yes	No	Yes	No	No	No	Yes	No
Choledocholithiasis	No	No	No	No	No	No	No	Yes	No	No	No
Treatment	Endoscopic retrograde gallbladder drainage	Percutaneous transhepatic gallbladder drainage	Laparoscopic cholecystectomy	Laparoscopic cholecystectomy	Cholecystectomy	Cholecystectomy	Cholecystectomy	Endoscopic retrograde biliary drainage	Cholecystectomy	Endoscopic biliary stenting	Antibiotics

*Af* atrial fibrillation, *AV* atrioventricular

It was found that 5 (45 %) of the 11 patients who developed cholecystitis had a recent history of stay in the ICU before the occurrence of cholecystitis. Two patients (cases 12 and 14) were diagnosed to have cholecystitis at the time of hospitalization in the cardiology department. One of them (case 14) had undergone endovascular vascular repair (coil embolization for internal iliac artery aneurysm) at the cardiothoracic surgery department and was staying in ICU. Because the patient was known to have coronary artery lesions, she was transferred to the cardiology department at post-operative day 12; at this time point, the patient had elevated hepatobiliary data (AST, 300 IU/L; Al-P 2086 IU/L) and was diagnosed to have cholecystitis. Another patient (case 12) was admitted to the cardiology department due to worsening of congestive heart failure, for which inflammation of the gallbladder was thought to aggravate the condition.

Two other patients were diagnosed with cholecystitis within a week of admission to the cardiology department (cases 8 and 16). Case 8 was diagnosed with cholecystitis on the fourth day of admission. This patient had been an in-patient for 12 days at another hospital until 2 days before their admission to our hospital. In case 16, cholecystitis was incidentally found by abdominal CT; an elevation in transaminase levels was not observed before the diagnosis, and the patient was treated by antibiotics and parenteral alimentation. Six (55 %) of the 11 patients in group 2 did not have any oral intake for ≥3 days, and five patients had been staying in ICU.

### Treatments

Among all 16 patients, 2 were treated with antibiotics, and the remaining 14 required invasive treatments including gallbladder resection (Tables 1, 4). In some patients, gallbladder drainage prior to the cholecystectomy was performed.

## Discussion

We identified 16 patients who were diagnosed with cholecystitis during hospitalization in the cardiology department. Among two patients, in which acute coronary syndrome was initially suspected, ECG changes suggestive of myocardial ischemia were observed. In eleven patients, in whom indeed had cardiovascular problems existed, eventually experienced gallbladder inflammation during hospitalization or at the time of admission. Fourteen patients (88 %) required cholecystectomy and/or gallbladder drainage for the treatment of cholecystitis.

It is well known that not only chest symptoms but also ECG changes suggestive of myocardial ischemia might be present in patients with cholecystitis, such as T-wave inversion, ST segment depression, and ST segment elevation [2, 10]. In addition to their confusing symptoms and ECG changes, H-FABP—a biochemical diagnostic marker in the early phase of acute myocardial infarction [11]—was positive in three (75 %) of four patients who were initially suspected to have acute coronary syndrome in group 1. H-FABP is known to be expressed also in non-cardiac organs, and it is elevated in non-cardiac disorders such as pulmonary embolism, and hepatic injury [12]. In addition, the H-FABP test, as well as cardiac troponin T, may show false-positive results in subjects with reduced renal function owing to its lower renal clearance [13, 14], but all three patients with positive H-FABP test had normal range renal function. All 3 patients with a positive H-FABP result, the cardiac troponin T test was negative. A previous case report showed elevation of troponin I and creatinine kinase levels in a patient who had acute cholecystitis but normal coronary arteries [4]. To the best of our knowledge, the present paper is the first to demonstrate that an H-FABP test may be positive in patients with cholecystitis.

In the patients in group 2, acute cholecystitis coexisted or developed with true cardiovascular problems. This might be just co-incidental; however, there were several possible reasons for coexistence of the two conditions. First, heart failure might worsen in the presence of gallbladder inflammation, which may have been the case for two patients (case 12 and 16). Second, acute cholecystitis can occur in critically ill patients. For, example, Laurila et al. reported that 39 (1 %) of 3984 patients staying in ICU developed acalculous cholecystitis [15]. In the current study, cholecystitis developed in five patients after or while staying on the ICU; although two cases were *calculous*.

The ECG changes associated with acute cholecystitis, when present at all, may resolve either after cholecystectomy or after a few days of antibiotic treatment [10]. Although the mechanisms by which acute cholecystitis causes ECG changes remain unclear, several possibilities have been suggested. First, distention of the bile duct may actually reduce the coronary artery flow [16, 17]. Second, there may be a coronary vasospasm that is caused by a vagally mediated reflex [2]. ECG changes may also be evoked in response to inflammation of other visceral organs; for instance, Rubio-Tapia reported that more than half of their study patients with acute pancreatitis showed certain ECG abnormalities, some of which might reflect electrolyte alterations and dehydration [18]. Furthermore, Yamazaki et al. reported a 53-year-old woman with chronic hepatitis C who showed ECG changes that mimicked myocardial ischemia [19].

There are several limitations in the current study. First, cardiac patients in general do not undergo screening by abdominal CT/US even in the presence of mild hepatobiliary data abnormalities; therefore, clinically silent or less severe cholecystitis might have been overlooked. Second, patients who experienced acute cholecystitis after their discharge were not counted. These conditions may have led to an underestimation of the prevalence of cholecystitis among in-patients in the cardiology department. Third, retrospective nature of the study. Subjects who have both cardiovascular disorders and acute cholecystitis might be found if we perform more complete survey by the prospective study. Fourth, small sample size of the study. We were unable to statistically demonstrate the patient characteristics that are more prone to be misdiagnosed to have cardiovascular disease. Fifth, incompleteness of the surveillance of the hepatobiliary system. The diagnosis was in general made by gastroenterologist; however, those with acute cholecystitis who had only mild or subclinical symptoms may not be diagnosed with cholecystitis because we did not decide to consult gastroenterologists.

## Conclusions

Among 5552 patients who were admitted to the cardiology department at our institute, we identified 16 patients who were diagnosed with acute cholecystitis during hospitalization—five patients who were initially suspected to have cardiovascular disease, which was later turned out not to be correct, and the remaining 11 patients were found to have both acute cholecystitis and cardiovascular diseases. Patients with acute cholecystitis may demonstrate, albeit rarely, ECG changes and laboratory abnormalities (such as positive H-FABP) that are suggestive of coronary ischemia; however, considering that we could diagnosed acute cholecystitis within 1 day after the admission, careful physical examination as well as routine laboratory tests of the hepatobiliary systems may able to avoid the delay of diagnosis of this gastroenterological disorder that might cause critical complications.
